# Comparison of risk scores for predicting adverse outcomes in acute lower gastrointestinal bleeding

**DOI:** 10.1016/j.heliyon.2024.e38877

**Published:** 2024-10-09

**Authors:** Chenyang Li, Ningning Zhang, Yuying Zhang, Nan Guo, Xiaomeng Sun, Shuling Li, Yan Xu, Tao Wang, Chao Chen

**Affiliations:** a Department of Gastroenterology, The First Medical Center of Chinese PLA General Hospital, Beijing, China; b Department of Gastroenterology, The Fourth Medical Center of PLA General Hospital, Beijing, China

**Keywords:** Gastrointestinal hemorrhage, Lower gastrointestinal tract, Adverse events, Risk stratification

## Abstract

**Objective:**

Acute lower gastrointestinal bleeding is a common emergency in gastroenterology. Currently, there is insufficient information to predict adverse outcomes in patients with acute lower gastrointestinal bleeding. Our study aimed to compare the effectiveness of the clinical risk scores currently utilized and their ability to predict significant outcomes in lower gastrointestinal bleeding.

**Methods:**

We conducted a prognostic study of patients hospitalized for acute lower gastrointestinal bleeding who underwent colonoscopy or angiography at a single-center hospital between January 2015 and October 2023. Adverse outcomes associated with ALGIB included rebleeding, blood transfusion, hemostatic interventions, and in-hospital death. We calculated three risk scores at admission (Oakland, Birmingham, SALGIB). We measured the accuracy of these scores using the area under the receiver operating characteristic curve (AUC) and compared them with DeLong's test.

**Results:**

222 patients with confirmed lower gastrointestinal bleeding (aged 64 years, 53–75) were finally included. The most common diagnoses were colorectal cancer (28 %) and hemorrhoids (14 %). The Oakland score, Birmingham score, and SALGIB score displayed comparable performance in predicting any adverse outcome (AUC = 0.54, 0.53, 0.55). However, none of the scores were able to sufficiently discriminate rebleeding, blood transfusion, or hemostatic intervention. Using the Youden index, cutoff points for predicting undesired results were identified for the Oakland score at 13, Birmingham score at 3, and SALGIB score at 2.

**Conclusions:**

None of the three scores demonstrated satisfactory discrimination for adverse outcomes. Therefore, it is necessary to develop novel risk stratification scores with higher performance to improve risk stratification in acute lower gastrointestinal bleeding.

## 1 Introduction

Acute lower gastrointestinal bleeding (ALGIB) stands as an urgent and potentially fatal clinical scenario, consistently accounting for a significant segment of gastroenterological emergencies [1,2]. The epidemiological landscape of gastrointestinal hemorrhage has been transforming, with a noted decrease in the incidence of upper gastrointestinal bleeding (UGIB) juxtaposed against a subtle yet steady rise in cases of ALGIB. In the United States, this trend is exemplified by a decline in UGIB rates from 112.3 to 94.4 per 100,000 between 2006 and 2014, counterbalanced by an increase in ALGIB from 146.0 to 161.0 per 100,000 over the same period [3]. While the majority of ALGIB episodes can be addressed through non-invasive measures, a considerable percentage of patients exhibit severe hemorrhage that demands intensive interventions, including blood transfusions and hemostatic procedures [2].

In the quest to better triage and manage patients with ALGIB, a variety of risk assessment tools have been conceptualized [[4], [5], [6], [7], [8], [9], [10], [11], [12]]. These instruments aim to pinpoint individuals at an elevated risk of adverse outcomes due to the severity of their bleeding. However, in contrast to the well-established risk scores for UGIB, no such consensus exists for the lower gastrointestinal counterpart. The variability in outcome metrics reported across different studies has posed challenges in the direct comparison and validation of these risk assessment models. Moreover, certain scoring mechanisms necessitate intricate clinical data that may not be promptly accessible in the emergent care environment, thus potentially undermining their prognostic accuracy concerning critical endpoints such as recurrent hemorrhage, the imperative for hemostatic interventions, and the necessity of blood transfusions.

The imperative for clinicians is to possess dependable prognostic indicators capable of precisely delineating the risk profile of patients with ALGIB, given the condition's inherent heterogeneity and the spectrum of treatment strategies contingent upon the degree of hemorrhage. This investigation seeks to undertake a critical evaluation and comparative analysis of the prognostic utility of several clinically recognized risk scores, namely the Oakland, Birmingham, and SALGIB scores, concerning their predictive accuracy for adverse outcomes encompassing rebleeding episodes, requisite for blood transfusions, need for hemostatic interventions, and overall clinical deterioration in individuals presenting with ALGIB.

## 2 Methods

### 2.1 Setting and participants

A single-center retrospective observational study was conducted in a tertiary center and teaching hospital. This study was approved by the Biomedical Research Ethics Committee of the Fourth Medical Center of Chinese PLA General Hospital(No. S2024-011-03), and patient consent was waived due to the retrospective design of the study. Patients aged ≥18 years with symptoms suggestive of overt ALGIB (i.e., red or maroon stool, blood mixed with stool, clots per rectum, or passage of melena without hematemesis) who were admitted and underwent colonoscopy or angiography from January 2015 to October 2023 were recruited. Exclusion criteria included age <18 years, LGIB in patients already hospitalized, confirmed UGIB source, unknown origin or lack of clinical records including endoscopy or angiography.

### 2.2 Data collection

Baseline demographics, clinical data (including preexisting medical conditions, vital signs, laboratory results, and prescribed medications), hospital management details, and adverse events (such as rebleeding, blood transfusions, hemostatic interventions, and in-hospital mortality) were extracted from electronic medical records. Endoscopy or angiography was performed depending on individual clinical practice and recorded as the primary procedure. Endoscopy reports were reviewed to determine the source of bleeding. Only definite sources of bleeding were included, defined as lesions with stigmata of recent bleeding(i.e., active bleeding, a visible vessel, or adherent clot), friable tumors or colitis [2].

### 2.3 Outcomes

The study revealed that the adverse consequences of ALGIB consisted of persistent bleeding within the first 24h and/or rebleeding, blood transfusion, hemostatic intervention, and in-hospital mortality. Rebleeding was defined as a recurrence of clinically significant bleeding that needed additional blood transfusion, repetition of endoscopic or radiologic procedures, or a further decrease in hematocrit by 20 % or more within 24 h after initial presentation. Blood transfusion was decided according to the recommendations of the National Institute for Health and Care Excellence [13]. The hemostatic intervention was a combination of surgical, endoscopic, and radiologic interventions. In-hospital mortality included deaths attributed to uncontrollable bleeding and severe comorbidities.

### 2.4 Statistical analysis

The numerical presentation was used for categorical data, whereas quantitative data underwent normality testing via the Kolmogorov‒Smirnov test, with nonnormally distributed data presented as the median and interquartile range. Comparisons across groups utilized the Fisher exact test, while continuous data were analyzed through 2-sample t-tests. The Oakland, Birmingham, and SALGIB scores were calculated from the data at admission (Online Resource Table 1). Sensitivity, specificity, and Youden index were calculated to detect the optimal cutoff point. The predictive performance in patients with LGIB was assessed by calculating the AUROC of each scoring system to detect any adverse outcomes [14]. The performance of AUROCs was compared between scoring systems using DeLong's test [15]. P < 0.05 was set for statistical significance. All statistical analyses were conducted using version 22.0 of SPSS software (IBM Corporation, Armonk, NY, USA).

**Table 1:** Demographic and clinical characteristics of patients in the study.

Characteristic	Total n = 222	No adverse outcome n = 119	Any adverse outcome n = 103	p-value
Age,median(IQR)	64(53,75)	64 (55, 75)	63(51, 74)	0.415
Sex				
Male,n(%)	129(58.1)	66 (55.5 %)	63 (61.2 %)	0.390
Previous admission with LGIB,n(%)	69(31.1)	28 (23.5 %)	41 (39.8 %)	**0.009**
Comorbidities				
Heart disease	52(23.4)	33 (27.7 %)	19 (18.4 %)	0.103
Stroke	26(11.7)	13 (10.9 %)	13 (12.6 %)	>0.999
Pulmonary disease	4(1.8)	2 (1.7 %)	2 (1.9 %)	>0.999
Liver disease	6(2.7)	1 (0.8 %)	5 (4.9 %)	0.099
Renal disease	11 (5.0 %)	6 (5.0 %)	5 (4.9 %)	0.949
Hypertension	94 (42.3 %)	45 (37.8 %)	49 (47.6 %)	0.142
Diabetes	38 (17.1 %)	15 (12.6 %)	23 (22.3 %)	0.055
Cancer	26 (11.7 %)	12 (10.1 %)	14 (13.6 %)	0.418
Past medical history of colorectal polyps	14 (6.3 %)	9 (7.6 %)	5 (4.9 %)	0.408
Preadmission medications				0.006
Aspirin	20 (9.0 %)	16 (13.4 %)	4 (3.9 %)	
Clopidogrel	9 (4.1 %)	7 (5.9 %)	2 (1.9 %)	
Dual antiplatelet	4 (1.8 %)	2 (1.7 %)	2 (1.9 %)	
Warfarin	4 (1.8 %)	2 (1.7 %)	2 (1.9 %)	
NSAIDs	4 (1.8 %)	0 (0.0 %)	4 (3.9 %)	
Corticosteroid	3 (1.4 %)	3 (2.5 %)	0 (0.0 %)	
Presenting signs and syptoms				
Clear red bloody stool in the ED	71 (32.0 %)	28 (23.5 %)	43 (41.7 %)	**0.004**
Blood on DRE	67 (30.2 %)	34 (28.6 %)	33 (32.0 %)	0.575
Heart rate(bpm)	78 (71, 85)	77 (71, 82)	78 (72, 87)	0.077
SBP(mmHg)	130 ± 19	132 ± 18	128 ± 20	0.181
Laboratory data at admission				
White blood cell count(∗10⁹/L)	6.20 (4.70, 7.58)	6.20 (4.65, 7.50)	6.20 (4.70, 7.75)	0.449
Hemoglobin(g/L)	115 (85, 132)	120 (107, 137)	95 (71, 125)	**<0.001**
Hematocrit(%)	35 (27, 40)	37 (33, 41)	30 (23, 38)	**<0.001**
Platelet(∗10⁹/L)	209 (161, 259)	209 (161, 262)	208 (161, 256)	0.793
BUN(mmol/L)	4.82 (3.80, 6.30)	4.80 (3.90, 5.80)	4.90 (3.80, 6.80)	0.530
Creatinine(μmol/L)	70 (58, 83)	71 (58, 82)	69 (57, 85)	0.874
Albumin(g/L)	37.7 (34.0, 41.4)	38.9 (35.1, 41.9)	37.0 (30.1, 40.0)	**0.001**
INR	1.10 (1.00, 1.10)	1.00 (1.00, 1.10)	1.10 (1.03, 1.20)	**<0.001**

Data are n(%), median (IQR: interquartile range), and mean ± SD. NSAIDs: nonsteroidal anti-inflammatory drugs; ED: emergency department; DRE: digital rectal examination; LGIB: lower gastrointestinal bleeding; SBP: systolic blood pressure; BUN: blood urea nitrogen.

## 3 Results

### 3.1 Patient characteristics and adverse outcomes

**Fig. 1:**
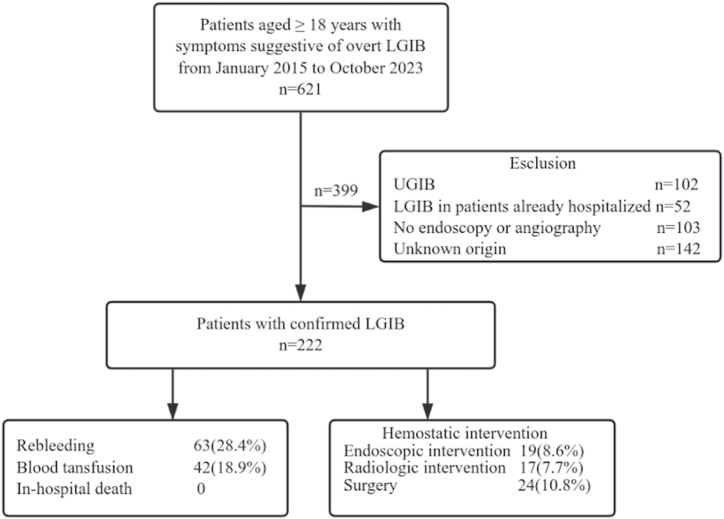
Flowchart of patients recruited for this study. ALGIB: acute lower gastrointestinal bleeding; UGIB: upper gastrointestinal bleeding.

**Fig. 2:**
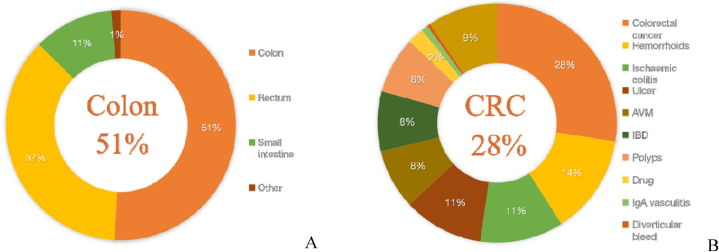
Sites and Sources of lower gastrointestinal bleeding. (A) Sites (B) Sources. CRC: colorectal cancer; AVM: arteriovenous malformation; IBD: Inflammatory bowel disease.

Over the course of our investigation, 222 individuals diagnosed with ALGIB were subjected to either colonoscopy or angiography. The median age of this patient population was 64 years, with a predominance of males at 58.1 %. A comprehensive overview of their demographic and clinical profiles is delineated in Table 1. Hypertension and heart disease emerged as the most prevalent comorbidities, affecting 42.3 % and 23.4 % of the cohort, respectively. A minority of patients were on antiplatelet therapy, with 9.0 % taking aspirin and 4.1 % on clopidogrel. Fig. 1 provides a detailed account of the adverse events associated with ALGIB during the study. The etiological factors behind ALGIB were elucidated through Fig. 2., revealing that in 80 % of the cases, a definitive cause was identified. Colorectal cancer was the leading etiology, accounting for 28 % of these cases, with hemorrhoids as the second most common source at 14 %. In terms of bleeding sites, 50.9 % of the cases involved the colon, while rectal hemorrhage was observed in 36.5 %. Throughout the hospital stay, no patient fatalities were recorded. However, a significant proportion of the cohort, 47 %, encountered at least one adverse event. Rebleeding was documented in 28.4 % of the patients, and 18.9 % required blood transfusions. Hemostatic interventions were necessary in several cases, with endoscopic procedures being performed in 8.6 %, radiological interventions in 7.7 %, and surgical interventions in 10.8 % of the patients.

### 3.2 Comparison of scoring systems in predicting adverse outcomes

**Fig. 3:**
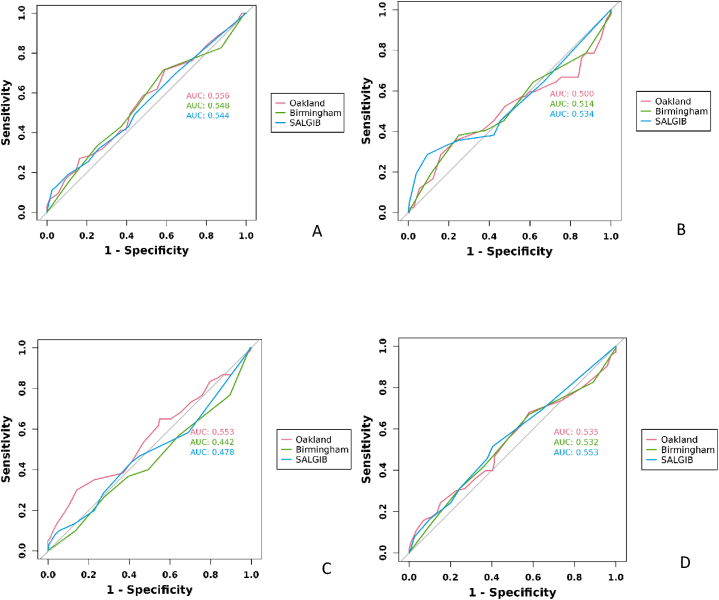
Receiver operating characteristic curves of each score for predicting rebleeding, blood transfusion, hemostatic intervention, and any adverse outcome. (A) Rebleeding (B) Blood transfusion (C) Hemostatic intervention (D) Any adverse outcome.

The Oakland score, Birmingham score, and SALGIB score displayed comparable performance in predicting any adverse outcome (AUC = 0.54, 0.53, 0.55) (Table 2, Fig. 3). However, none of the scores were able to sufficiently discriminate rebleeding, blood transfusion, or hemostatic intervention. Using the Youden index, cutoff points for predicting undesired results were identified for the Oakland score at 13, Birmingham score at 3, and SALGIB score at 2 (Table 3).

**Table 2:** Performance of different risk scores in comparison with the Oakland score in the prediction of adverse outcomes.

	Rebleeding	Blood transfusion	Hemostatic intervention	Any adverse outcome
Oakland	n = 63(28.4 %)	n = 42(18.9 %)	n = 60(27.0 %)	n = 103(46.4 %)
	0.556(0.47–0.64)	0.50(0.39–0.61)	0.55(0.46–0.64)	0.54(0.46–0.61)
Birmingham	0.548(0.46–0.63)	0.51(0.41–0.62)	0.44(0.35–0.53)	0.53(0.46–0.61)
	p = 0.714	p = 0.895	p = 0.205	p = 0.865
SALGIB	0.54(0.46–0.63)	0.53(0.43–0.64)	0.48(0.39–0.57)	0.55(0.48–0.63)
	p = 0.673	p = 0.741	p = 0.364	p = 0.492

Data are presented as areas under the receiver operating characteristic curve and 95 % confidence intervals; p values are from the Delong et al. test.

**Table 3:** Optimal cutoff value of different scores for predicting the presence of any adverse outcome.

Score	Cutoff value	Sensitivity	Specificity	PPV	NPV	Youden index
Oakland	12	73.79 %	26.05 %	46.34 %	53.45 %	−0.002
	**13**^a^	67.96 %	42.02 %	50.36 %	60.24 %	0.100
	14	61.17 %	46.22 %	49.61 %	57.89 %	0.074
Birmingham	2	82.52 %	10.92 %	44.50 %	41.94 %	−0.066
	**3**^a^	66.99 %	42.02 %	50.00 %	59.52 %	0.090
	4	50.49 %	56.30 %	50.00 %	56.78 %	0.068
SALGIB	1	68.93 %	36.13 %	48.30 %	57.33 %	0.051
	**2**^a^	51.46 %	59.66 %	52.48 %	58.68 %	0.111
	3	45.63 %	62.18 %	51.09 %	56.92 %	0.078

PPV: positive predictive value; NPV: negative predictive value.

a Cutoff points determined by the Youden index.

## 4 Discussion

Lower gastrointestinal hemorrhage frequently presents as an urgent clinical challenge in gastroenterology units. A plethora of predictive models have been developed to identify patients who may experience significant bleeding episodes. Yet, the sheer number of these models can complicate the process of selecting the most suitable ones for clinical application. In contrast, for UGIB, a selection of accessible and straightforward risk scores is readily available to assist clinicians in decision-making and to optimize patient outcomes [16]. In this study, we conducted a comparative analysis of a range of risk assessment instruments to ascertain their efficacy in foreseeing untoward outcomes.

The incidence of adverse effects observed in our study paralleled findings from Tapaskar et al. in the United States [17] but was notably higher than rates reported in other cohorts [8,13,18,19]. This discrepancy may be attributed to the inclusion of only confirmed cases of LGIB and the exclusion of low-risk patients discharged from the emergency department. Notably, no in-hospital mortality was recorded during the study period, likely due to the efficacious hemostasis treatments administered to patients with severe bleeding and the exclusion of patients with critical conditions who could not undergo endoscopic or angiographic evaluations. Our findings underscore the significant rebleeding rates associated with ALGIB, which are considerably higher than those reported in other population-based studies [8,[20], [21], [22]]. In our cohort, colorectal cancer emerged as the predominant cause of LGIB, corroborating the results of Bai et al. in China [23]. This finding contrasts with the prevalent cause of diverticular bleeding observed in Western populations [24,25]. However, the prevalence of diverticular bleeding, as reported by Quach et al., ranges from 6.0 % to 8.7 % in the Asian population, highlighting the need for further research to elucidate these demographic differences [2,13].

The Oakland score has been extensively validated for the assessment of LGIB. It is recommended by clinical guidelines in the United States and the United Kingdom as a primary tool for patient triage [24,26]. Oakland et al. tested the predictive ability of the Oakland score in a validation set of 288 patients and found that it performed well in predicting safe discharge (AUC = 0.84), rebleeding (AUC = 0.74), and transfusion (AUC = 0.92). In 2020, Oakland et al. conducted an external validation study of 38,067 patients, demonstrating good performance in predicting safe discharge (AUC = 0.87) and transfusion (AUC = 0.90) [27]. Patients with an Oakland score ≤8 had a sensitivity of 98.4 % and a specificity of 16.0 % for safe discharge, with 1.1 % experiencing in-hospital mortality and 7.5 % having adverse outcomes. This study fully demonstrated the power of the Oakland score in predicting severe lower gastrointestinal bleeding. In an external validation cohort of 761 patients, Mostafa et al. found that the Oakland score accurately predicted adverse events (AUC = 0.85), with a 95 % probability of safe discharge for patients with an Oakland score ≤10 [28]. The SALGIB score, introduced more recently in Vietnam, has shown comparable predictive accuracy for severe bleeding to the well-established Oakland score [13]. Quach DT et al. demonstrated the good predictive ability of the SALGIB score for severe lower gastrointestinal bleeding in a validation set of 324 patients, with AUC of 0.86. In 2021, Quach DT et al. further confirmed the predictive ability of the score for severe lower gastrointestinal bleeding in an external validation study of 414 patients, achieving an AUC of 0.87. The score also showed good predictive ability for transfusion (AUC = 0.91) and mortality (AUC = 0.82) [2,13]. In addition, our previous study demonstrated the strong ability of the SALGIB score to predict transfusion (AUC = 0.95) and major lower gastrointestinal bleeding (AUC = 0.78) [1]. Our study, however, did not identify any single score that excelled across all evaluated outcomes. The Birmingham score, developed in the United Kingdom, incorporates fewer parameters, enhancing its user-friendliness [9]. Both the SALGIB and Birmingham scores, due to their streamlined components, offer greater practicality for routine clinical use compared to the more complex Oakland scores. Other scoring systems are in their nascent stages and lack robust external validation data. Regrettably, none of the three scores provided satisfactory predictive accuracy for any adverse outcome.

It is imperative to acknowledge the limitations inherent in our study. As a retrospective, single-center investigation, our findings may not be generalizable to hospitals with differing patient demographics and disease severities. The modest sample size may have limited our ability to identify specific variables or scores with reliable predictive power for adverse events. The study's criteria focused exclusively on patients who underwent colonoscopy, which could introduce selection bias. Furthermore, patients who were discharged from the emergency department or were ineligible for endoscopic or angiographic procedures were not included, thereby constraining our capacity to fully assess the clinical utility of these scoring systems. Future research should encompass multicenter, prospective studies to more accurately compare the efficacy of these risk scores in identifying patients at high and low risk for ALGIB.

## 5 Conclusions

In summary, our comparative analysis of multiple risk scores revealed that none provided exceptional predictive accuracy for adverse outcomes in ALGIB. There is a clear need for the development of novel risk stratification scores that offer superior predictive performance to enhance the precision of risk assessment in ALGIB.

## CRediT authorship contribution statement

**Chenyang Li:** Writing – original draft, Formal analysis, Conceptualization, Data curation, Funding acquisition, Investigation, Methodology, Project administration, Resources, Software, Validation, Visualization. **Ningning Zhang:** Writing – original draft, Methodology, Conceptualization, Data curation, Formal analysis, Funding acquisition, Investigation, Project administration, Resources, Software, Validation, Visualization. **Yuying Zhang:** Writing – review & editing, Conceptualization, Supervision. **Nan Guo:** Writing – review & editing, Conceptualization, Supervision. **Xiaomeng Sun:** Writing – review & editing, Conceptualization, Supervision. **Shuling Li:** Writing – review & editing, Conceptualization, Supervision. **Yan Xu:** Writing – review & editing, Conceptualization, Supervision. **Tao Wang:** Writing – review & editing, Conceptualization, Supervision. **Chao Chen:** Writing – review & editing, Conceptualization, Supervision.

## 7 Data availability statement

The data associated with the study has not been deposited into a publicly available repository. Data will be made available on request.

## 8 Ethics approval and consent to participate

Ethical approval was waived by the local Ethics Committee of the Fourth Medical Center of Chinese PLA General Hospital in view of the retrospective nature of the study and all procedures being performed were part of the routine care.

## 6 Funding

Authors did not receive support from any organization for the submitted work.

## Declaration of competing interest

The authors declare that they have no known competing financial interests or personal relationships that could have appeared to influence the work reported in this paper.
